# Supplementary material for: Levofloxacin Cocrystal/Salt with Phthalimide and Caffeic Acid as Promising Solid-State Approach to Improve Antimicrobial Efficiency
Source: Antibiotics (Basel). 2022 Jun 13;11(6):797. doi: 10.3390/antibiotics11060797 (PMC9220774; doi:10.3390/antibiotics11060797)
Supplement: Supplementary file 1 [file antibiotics-11-00797-s001.zip › antibiotics-1747963-supplementary.pdf]

Supplementary File

Levofloxacin Cocrystal/Salt with Phthalimide and Caffeic Acid as Promising Solid-State Approach to Improve Antimicrobial Efficiency

Noor Ul Islam [1], Muhammad Naveed Umar [1], Ezzat Khan [1,2], Fakhria A. Al-Joufi [3], Shaymaa Najm Abed [4],
Muhammad Said [1], Habib Ullah [5], Muhammad Iftikhar [6], Muhammad Zahoor [6,*] and Farhat Ali Khan [7]

[1] Department of Chemistry, University of Malakand, Chakdara, Dir Lower 18800, Khyber Pakhtunkhwa, Pakistan; nooruomchem@gmail.com (N.U.I.); m.naveedumar@uom.edu.pk (M.N.U.); ekhan@uom.edu.pk (E.K.); msaidqau@yahoo.com (M.S.)
[2] Department of Chemistry, College of Science, University of Bahrain, Sakhir 32038, Bahrain
[3] Department of Pharmacology, College of Pharmacy, Jouf University, Aljouf 72341, Saudi Arabia; faaljoufi@ju.edu.sa
[4] Nursing Department, College of Applied Medical Sciences, Jouf University, Sakaka 72311, Saudi Arabia; snabed@ju.edu.sa
[5] Department of Nuclear and Quantum Engineering, Korea Advanced Institute of Science and Technology (KAIST), Daejeon 34141, Korea; habibuom24@gmail.com
[6] Department of Biochemistry, University of Malakand, Chakdara, Dir Lower 18800, Khyber Pakhtunkhwa, Pakistan; miftikhar355@gmail.com
[7] Department of Pharmacy, Shaheed Benazir Bhuto University, Sheringal, Dir Upper 18000, Khyber Pakhtunkhwa, Pakistan; farhatkhan2k9@yahoo.com
* Correspondence: mohammadzahoorus@yahoo.com

Construction of binary phase diagram

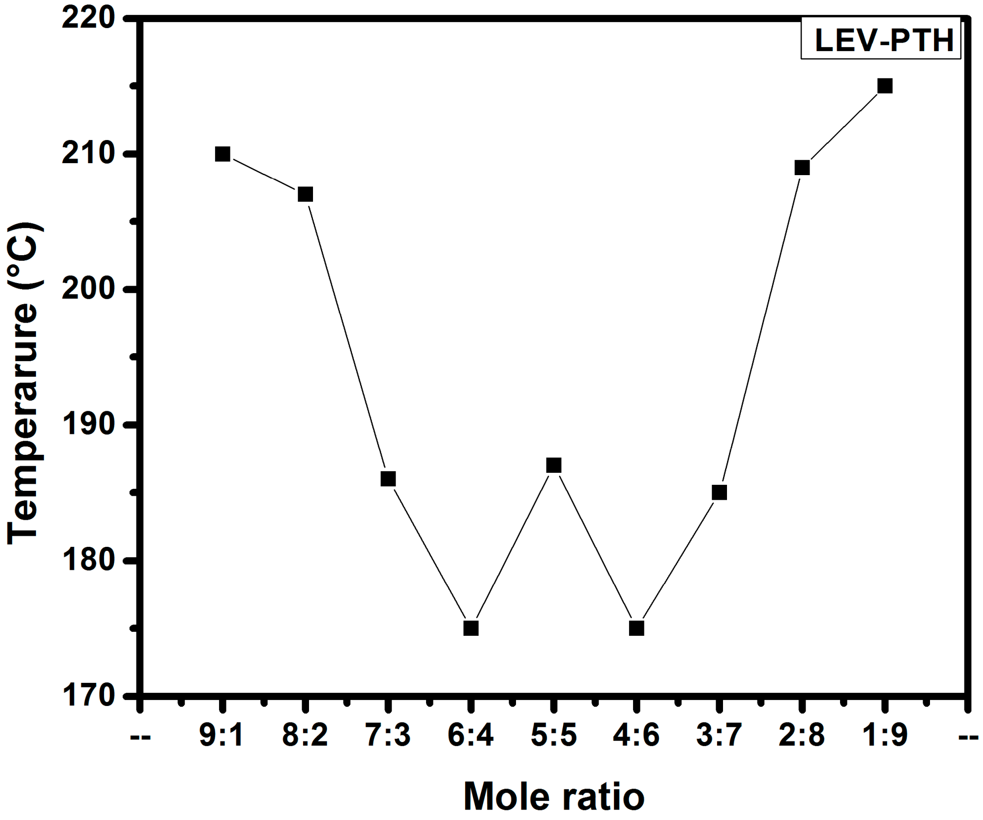

Figure S1. Binary phase diagram for LEV and CFA in different molar ratios.

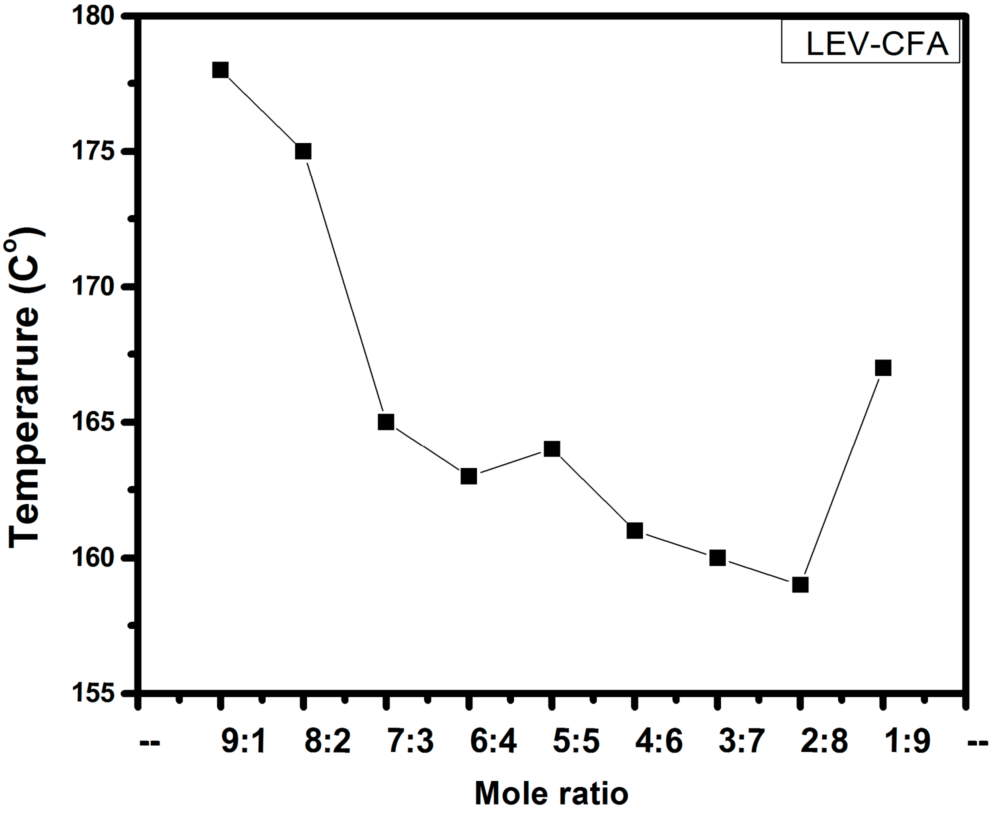

Figure S2. Binary phase diagram for LEV and CFA in different molar ratios.

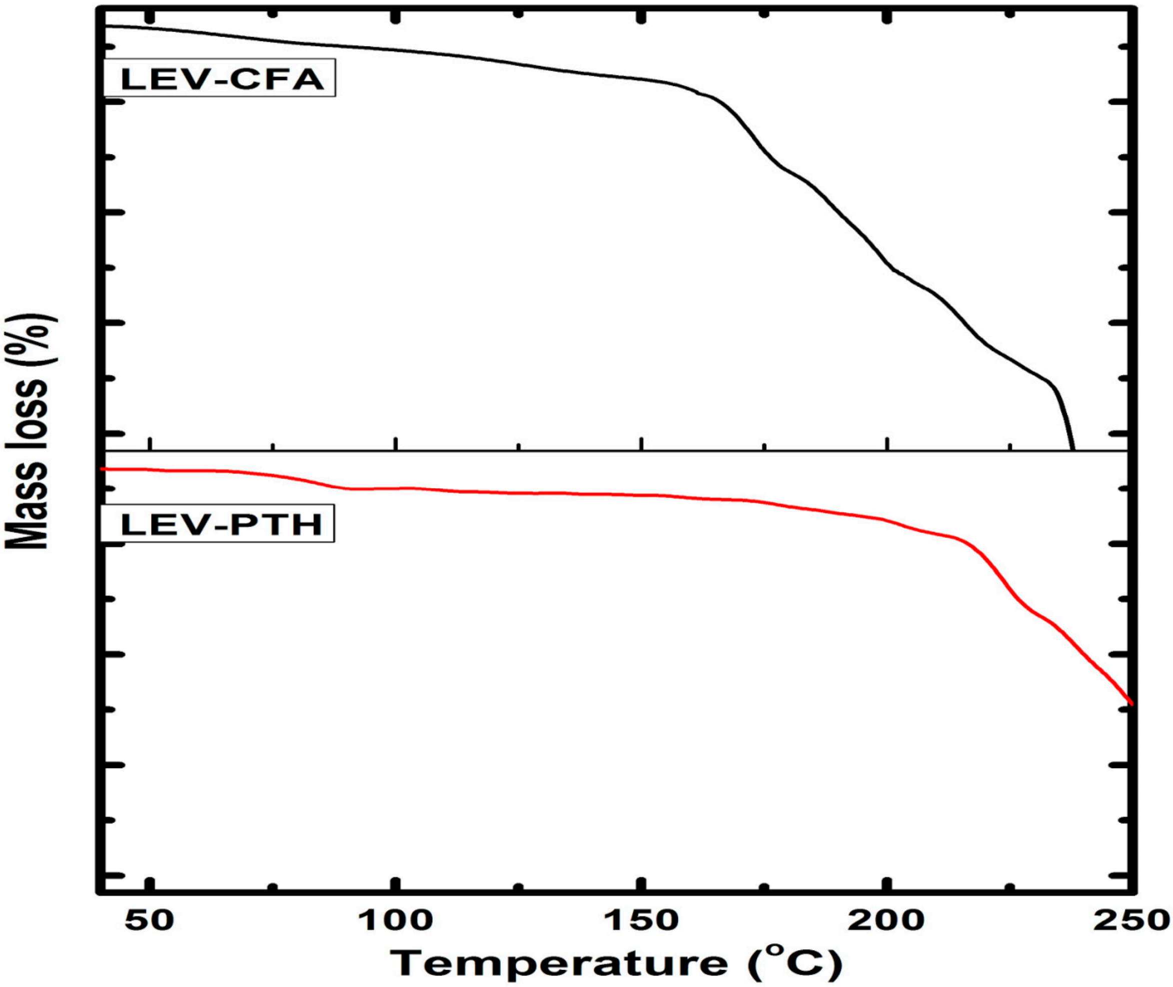

Figure S3. TG thermograms of cocrystal (LEV-PTH) and amorphous salt (LEV-CFA).

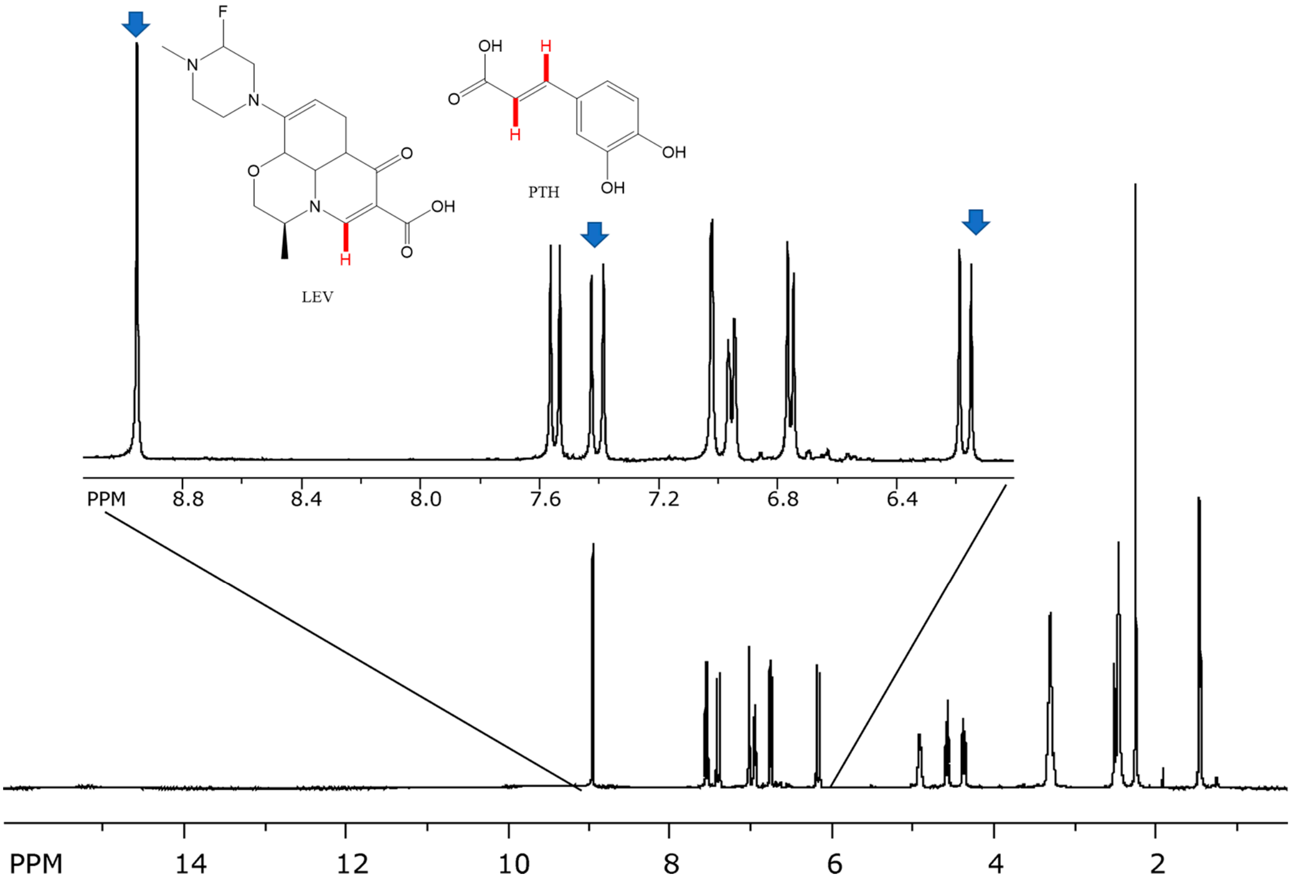

Figure S4. ^{1}H-NMR (400 MHz, in $CDCl_3$) spectra of LEV-PTH, integrated protons (red in individual Chemdraw structures) showing their presence in 1:1 ratio are marked by arrows. NMR Chemical Shift values (s ppm, $^nJ(^1H,^1H)$ in Hz) = 15.18 br, 8.95 s, 7.56-7.53 d (12.3 Hz), 7.42 – 7.39 d (15.9 Hz), 7.02 s, 6.96 – 6.95 d (7.8 Hz), 6.76 – 6.74 d (8.0 Hz), 6.19-6.15 d (15.9 Hz), 4.91 q, 4.59 – 4.56 d (11.5 Hz), 4.38 – 4.35 d (11.5 Hz), 3.30 q, 2.45, 2.24, 1.45 d(6.7 Hz).

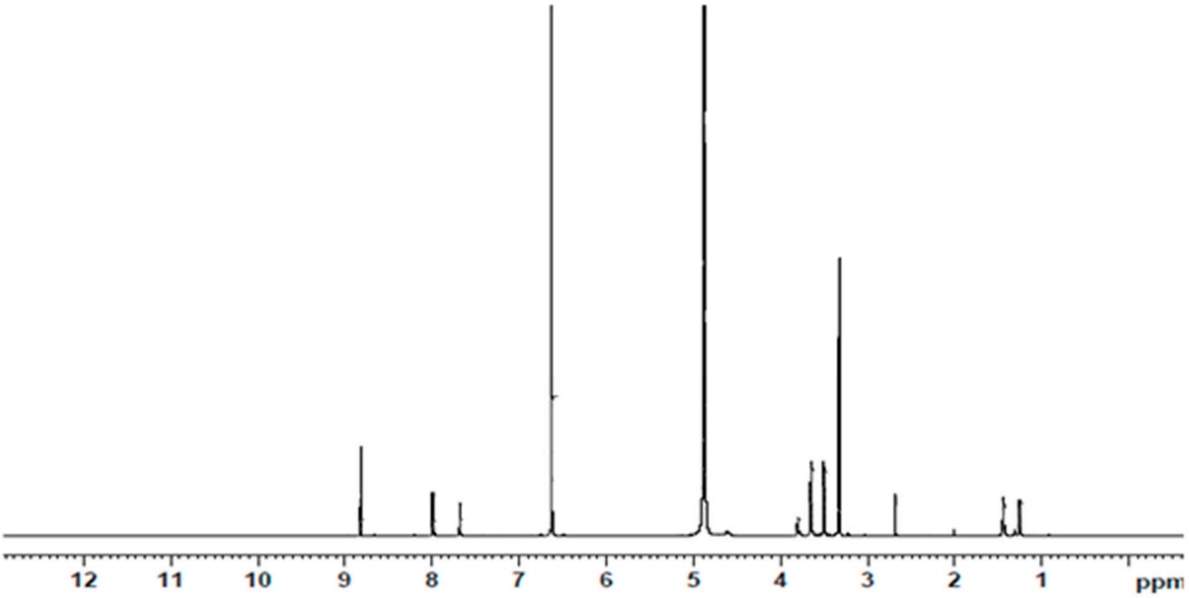

Figure S5. ^{1}H-NMR (400 MHz) spectra of LEV-CFA. NMR Chemical Shift values (s ppm, $^{n}J(^{1}H,^{1}H)$ in Hz) = 8.82 s, 8.00-7.98 d, 7.69, 4.88 s, 3.82, 3.79, 3.65, 3.50, 1.45, 1.45.

Micrometric study

Micrometric analysis of parental drug and prepared cocrystal/salt were performed and the obtained results were displayed in table S2.

Bulk density (BD)

Specific amount of powders LEV and cocrystal/salt were subjected in a measuring volume cylinder and noted bulk volume of each sample. Bulk density (BD) of cocrystal/salt and respective drug was calculated using the following formula:

$$BD = \frac{\text{mass of the sample}}{\text{bulk volume of the sample}}$$

Tapped density (TD)

After measuring bulk volume, the cylinder was tapped 100 times from height of 2 cm and volume of LEV, cocrystal and salt were calculated using the following formula:

$$TD = \frac{\text{mass of the sample}}{\text{tapped volume of the sample}}$$

Carr's index (compressibility index)

Compressibility parameter of LEV, cocrystal and salt were calculated as below:

$$\text{Compressibility index (CI)} = \frac{TD - BD}{TD} \times 100$$

Hausner's Ratio (HR)

The Hausner's ratio for the drug, cocrystal and salt were calculated using the formula:

$$HR = TD/BD$$

Table S1. Flow properties of LEV, cocrystal and salt.

Flow property	LEV	Cocrystal (LEV-PTH)	**Salt (LEV-CFA)**
**Bulk volume (mL)**	1.2		1.5
**Bulk density (g/mL)**	0.83		0.67
**Tapped volume (mL)**	0.80		1
**Tapped density (g/mL)**	1.25		1
**Compressibility index**	33.6		33
**Hausner's ratio**	1.50		1.49
